# Building the National Antimicrobial Resistance Surveillance Network in Animals in Greece: A “One Health” Approach

**DOI:** 10.3390/antibiotics12091442

**Published:** 2023-09-13

**Authors:** Christos Zafeiridis, George Valiakos, Panagiota Giakoupi, Emmanouil Papadogiannakis

**Affiliations:** 1 General Directorate of Veterinary Services, Ministry of Rural Development & Food of Greece, 10176 Athens, Greece; chzafeiridis@uniwa.gr; 2 Faculty of Veterinary Science, University of Thessaly, 43100 Karditsa, Greece; georgevaliakos@uth.gr; 3 Department of Public Health Policy, School of Public Health, University of West Attica, 11521 Athens, Greece; pgiakoupi@uniwa.gr

**Keywords:** antimicrobial resistance, diseased animals, EARS-Vet, One Health, veterinary medicine

## Abstract

It is widely accepted that, in order to prevent and control antimicrobial resistance (AMR), surveillance systems across human, animal and environmental sectors need to be integrated, in a One Health approach. Currently, in Europe, there are surveillance networks established only for the human and food sector and, until now, there has been no organized effort to monitor AMR in bacterial pathogens derived from diseased animals in Europe. Since 2017, efforts to fill this gap have taken place by the European Antimicrobial Resistance Surveillance network in a veterinary medicine (EARS-Vet) initiative, included in the EU Joint Action on AMR and Healthcare-Associated Infections (EU-JAMRAI). EARS-Vet is designed to complement and integrate with existing European monitoring systems for AMR as well as constitute a European network of national monitoring systems. As Greece has no national AMR surveillance system for pathogens of animal origin currently in place, in the context of the development of EARS-Vet, an initiative took place for the organization of such a system by competent agencies and other stakeholders. In this article, the steps to organize a first AMR national surveillance network in Greece are presented and a Strengths, Weaknesses, Opportunities, and Threats (SWOT) analysis is performed to present main characteristics of the approach implemented.

## 1. Introduction

Although significant efforts have been made in recent years to control the use of antibiotics, the Antimicrobial Resistance (AMR) public health problem is still considered significant, leading to millions of deaths worldwide and thousands in Europe every year [1]. In the European Union (EU), surveillance systems have been developed across human, animal and environmental sectors to monitor the current situation, in a One Health approach as stated in the EU Action Plan against AMR [2]. In the human sector, two surveillance systems are currently in place in the EU: one is called the European Antimicrobial Resistance Surveillance Network (EARS-Net). This system performs a surveillance of antimicrobial susceptibility of eight invasive bacterial pathogens isolated from blood or cerebrospinal fluid, commonly causing infections in humans [3]. Another system called European Food- and Waterborne Diseases and Zoonoses Network (FWD-Net) monitors AMR in human *Salmonella* and *Campylobacter* infections [4]. In the animal and food sector, the European Food Safety Authority (EFSA) coordinates an active monitoring of AMR in zoonotic (*Salmonella* and *Campylobacter*), indicator bacteria (*Escherichia coli*) and extended-spectrum-cephalosporin-resistant and carbapenemase-producing *E. coli* from healthy food-producing animals [5]. It is easily understood from the above that there is a significant lack of data regarding AMR in bacterial isolates derived from diseased animals, and thus, the role they may play in the spread of AMR to other animals, humans and the environment. Moreover, with no data from diseased animals, there cannot be any serious attempts to organize antimicrobials stewardship initiatives or propose specific treatment guidelines [6]. Having knowledge that, one of the key activities of the European Union (EU) Joint Action on AMR and Healthcare Associated Infections (EU-JAMRAI), was to strengthen an “One Health” AMR surveillance approach in Europe, the building of the European Antimicrobial Resistance Surveillance network in Veterinary medicine (EARS-Vet) was proposed by the EU Member States [7]. In the context of EU-JAMRAI, it was decided that EARS-Vet would work as a European network of national surveillance systems in diseased animals (similarly to the European Antimicrobial Resistance Surveillance Network in human medicine—EARS-Net) and should complement and integrate—under the One Health approach—with EARS-Net and the EFSA monitoring. Its objectives are to report on the current AMR situation surveying AMR trends in bacterial pathogens of animal origin in EU Member States (Greece included) [8]. This knowledge could be used for: (i) advising policy makers on how to intervene for mitigating AMR and monitoring the efforts to tackle AMR in the animal sector, (ii) supporting antimicrobial stewardship by developing antimicrobial treatment guidelines for the veterinary sector, (iii) generating/re-establishing missing epidemiological cut-off values and clinical breakpoints for the interpretation of antimicrobial susceptibility testing (AST) results, (iv) assessing the risk of AMR transmission from animals to humans via non-food related routes, e.g., by direct contact with food animals, and (v) estimating the burden of AMR in animal health, e.g., attributable deaths caused by infections with antimicrobial-resistant bacteria [8].

This manuscript outlines the approach employed to assess the feasibility of establishing an AMR surveillance system for diseased animals in Greece. The primary objective was to determine the extent to which such a monitoring system could be built, while also identifying the specific animal and bacterial species as well as the antimicrobial combinations that would be included in the initial phase of the surveillance system. A Strengths, Weaknesses, Opportunities, and Threats (SWOT) analysis is performed to highlight the advantages and disadvantages of the approach chosen and to be a source of information for similar initiatives in other countries.

## 2. Organization of a Pilot Project: Decisions and SWOT Analysis

During the first kick-off meeting (see Section 4), it was decided that, in order to evaluate the feasibility of establishing a monitoring system of AMR surveillance in diseased animals in a country without a system in place (i.e., Greece), a “pilot project” should be established as a first step. This pilot project should be based on similar systems already used in countries with an established monitoring system. Efforts to organize this pilot study would allow a SWOT analysis for the building of a broader AMR surveillance network in veterinary medicine by the relevant stakeholders. The Ministry of Rural Development & Food took the responsibility for the coordination of that initiative, defining a member of the National Action Plan (NAP) Steering Committee as the pilot project coordinator.

All information collected during the following meetings with relevant stakeholders, as well as during the 2020 presence meeting, led to the following conclusions.

The proposed scope for the pilot project is summarised in Table 1 in terms of animal species, production types, specimens and bacterial species included.

As regards initially proposed combinations that included *Klebsiella pneumoniae*, *Pseudomonas aeruginosa*, *Acinetobacter baumannii*, *Enterococcus faecalis* and *Enterococcus faecium*, that are monitored by EARS-Net in the human sector, it was decided not to include these in the Greek pilot project as even countries with established national surveillance systems reported that it was not feasible to provide AMR information in the EARS-Vet framework [7]. However, carbapenemase-producing *E. coli*, ESBL-producing *E. coli*, methicillin-resistant *S. aureus* and colistin-resistant *E. coli* would be covered in the proposed scope.

The proposed scope for the pilot project is summarised in Table 2 in terms of the antimicrobial combinations included, with some antimicrobial agents (e.g., enrofloxacin and ceftiofur) prescribed only for veterinary use but having a crucial role in AMR emergence.

The use of multiple antibiotics in veterinary medicine is necessary due to the diverse range of bacterial infections that affect animals. Veterinarians require a variety of antibiotics to effectively treat different infections and prevent the development of antibiotic resistance. The relevant specific indications of the antibiotics targeted in the pilot project are summarized in Table 3.

Describing the key areas in the Greek pilot project, as stated in Section 4, the following decisions were made and actions took place.

### 2.1. Description of the Key Areas in the Greek Pilot Project

#### 2.1.1. Political and Financial Support

By publishing a relevant Circular by the MRDF in March 2021, the Greek State confirmed that it strongly supports this initiative of AMR surveillance in pathogens derived from animals, but participation or data collection has not been defined as mandatory by any legislative framework. As it was highlighted by the EARS-Vet network, human and financial resources are frequent limitations of national AMR surveillance systems. Thus, funding needs, even for the narrow-scope pilot project, constitute a significant obstacle. To overcome this difficulty, and as this project would rely on data produced by relevant stakeholders around Greece, the livestock corporation representatives committed, during the initial meetings, that all material costs needed for initiating this pilot study, would be covered by their own funding.

#### 2.1.2. Monitoring Objectives

*Staphylococcus aureus* is a frequent colonizer of human and several animal species, including dairy cows. It is the most common cause of intramammary infections in dairy cows [9,10]. Its public health importance increases in line with the continuous emergence of drug-resistant strains, such as Methicillin-resistant *S. aureus* (MRSA). Indeed, the recent emergence of human and animal adapted MRSA demands serious attention. A main aim of this pilot project is to determine the burden and drug resistance pattern of *S. aureus* in dairy farms in all 13 Administrative Regions of Greece.

In addition, another aim is to investigate the presence of *Escherichia coli* at a stage after the end of the rearing period of piglets; post-weaning diarrhoea caused by *Escherichia coli* is considered one of the most important diseases in pig production worldwide with a huge economic impact [11]. Rectal samples are collected from all 13 Administrative Regions of Greece, in order to isolate the pathogen and characterise all isolates in terms of antibiotic resistance profiles, using the techniques described in the Laboratory Techniques key area (see below).

As regards the monitoring objectives which have been reported in various national monitoring systems in the EARS-Vet Network [8], the Greek pilot project targets to monitor AMR trends for the included bacterial and animal species, detect emerging AMR for antimicrobials of interest, as well as monitor AMR trends and detect emerging AMR in a public health perspective. As various stakeholders (veterinarians, livestock industry etc.) participate in the pilot project, collected information will be disseminated to them to help them in their antimicrobial treatment decisions.

#### 2.1.3. Central Institutional Organization

The Steering committee, as has been established by the Greek NAP, defines the vision and objectives of the monitoring system, as well as approves monitoring procedures; for the pilot project, the above are proposed by the Coordination Team. Coordinating institutions [private and public diagnostic laboratories and representatives of different livestock sectors (farmers, veterinarians etc.)], are defined as those running monitoring procedures in practice (i.e., interacting with data providers, collecting data, analysing data, writing reports etc.) under the stewardship of the National Antimicrobial Reference Laboratory—Department of Veterinary Laboratory of Chalkis—Directorate of Veterinary Center of Athens—Directorate General of Veterinary Services—Ministry of Rural Development & Food.

#### 2.1.4. Laboratory Network

For the pilot project, the laboratory network responsible for the AMR investigation of isolates includes one central (public sector, namely the National Antimicrobial Reference Laboratory—Department of Veterinary Laboratory of Chalkis—Directorate of Veterinary Centre of Athens—Directorate General of Veterinary Services—Ministry of Rural Development & Food) and one private sector diagnostic laboratory; due to financial restraints, for the pilot project, one private sector laboratory is responsible for AMR monitoring in samples from the targeted animal species (cattle—swine), with the prediction of expanding the network as soon as financial support is available; the central public sector laboratory will not perform re-testing due to the same financial constraints but will play an advisor role to the private network (if expanded) for the harmonization of the AST methods implemented.

#### 2.1.5. Monitoring Procedures—Monitoring Data

The pilot project is primarily based on an active monitoring procedure, where diseased animals (clinical and sub-clinical cases) are sampled specifically for the purpose of AMR monitoring and are submitted by field veterinarians to the private diagnostic laboratory. This active surveillance is considered to generate more representative AMR data than by passive monitoring; collecting passive data from veterinary diagnostic specimens tends to overestimate the AMR levels, as they are usually performed only after treatment failure [12]. For the moment, all monitoring data are sent to the pilot project coordinator (based at MRDF) and then it is progressively uploaded to WHONET [13], a free desktop Windows application for the management and analysis of microbiology laboratory data with a particular focus on antimicrobial resistance surveillance developed and supported by the WHO Collaborating Centre for Surveillance of Antimicrobial Resistance and coordinated (in Greece) by the National Public Health Organization (NPHO).

#### 2.1.6. Laboratory Techniques

Laboratory techniques and standards of the Greek pilot project are described as follows: Collection of samples is focused on dairy cows and post-weaning piglets that exhibit relevant clinical symptoms (mastitis and diarrhoea, respectively). All collected faecal and milk samples are transported in an icebox and investigation is initiated within 24 h. For swine faecal samples, a culture is performed immediately after receipt, to isolate *E. coli*. Specifically, in cases where faeces are collected directly from the rectum using disposable gloves and sterile containers, a quantity of faeces of 1 gr is diluted in a quantity of 9 mL of diluent MRD (Maximum Recovery Medium). Then, an inoculation takes place from the dilution to sheep blood agar and MacConkey agar and the plates are incubated at 37 °C for 18–24 h. In most cases, faecal samples are collected directly from the rectum using sterile cotton swabs; inoculation is performed directly on the agar plates. Three phenotypically different lactose-fermenting colonies from MacConkey agar and three different colonies from blood agar are isolated on Nutrient agar. After incubation for 18–24 h at 37 °C, the suspected *E. coli* colonies are tested for indole production at 44 °C in tryptone water broth. For bovine samples, culture is performed immediately after receipt, to isolate *Staphylococcus aureus*. Regarding collection procedure, milk from dairy cows is collected by pooling samples from all udder quarters of each lactating cow. The udders are cleaned thoroughly with water, dried using a clean towel, and the teat ends are disinfected with cotton swabs soaked in 70% alcohol, then left to air dry. After discarding the first streams of milk, three to four streams (1–2 mL each) from each udder quarter (totalling 12 streams from a single cow) are collected into sterile leak-proof plastic containers. In the laboratory, a quantity of 1 ml of milk is diluted in a quantity of 9 mL of MRD. Then, inoculation is performed from the original sample on a sheep blood agar plate as well as inoculation of 50 μL of both the original sample and the dilution using the spread plate technique on Mannitol Salt agar plates, for better recovery capacity of the target microorganism. All plates are incubated at 37 °C for 24–48 h. Three phenotypically different mannitol positive colonies from the Mannitol Salt agar plates and three different beta-haemolytic colonies from the blood agar, are isolated on Nutrient agar. After 18–24 h of incubation at 37 °C, the suspected *Staphylococcus aureus* colonies are Gram stained and tested for catalase production. Gram positive & catalase positive cocci are tested for coagulase production by tube method with coagulase plasma.

Finally, the indole-positive (*E. coli*) and the coagulase-positive (*S. aureus*) colonies are subjected to a Kirby–Bauer sensitivity test. Specifically, a suspension is prepared from the confirmed colonies in 0.9% NaCl to obtain a turbidity of 0.5 McFarland units. Using a sterile cotton swab, the suspension is spread on a Mueller Hinton agar plate and the appropriate antibiotic disks, as reported previously and according to Clinical and Laboratory Standards Institute (CLSI) standards, are placed. Regarding the AMR interpretation of results, the Greek pilot project follows mainly the CLSI standards and interprets results according to its veterinary clinical breakpoints (when available) [14,15]. When veterinary breakpoints are not available, epidemiological cut-off values (ECOFFs) of the European Committee on Antimicrobial Susceptibility Testing (EUCAST), Epidemiological Cutoff Values (ECVs) of CLSI and human clinical breakpoints of EUCAST or CLSI, are used. In the case of colistin, a published approach is applied for the use of the disc diffusion test [16]. It is important to note that, until harmonization on the AST method is reached among the participating EARS-Vet network countries, quantitative data (e.g., inhibition zone diameters) are accepted regardless of the standards under which they are produced [7]. All related isolates are kept for future Minimum Inhibitory Concentration (MIC) analysis.

#### 2.1.7. Communication

Data will be analysed, and the survey results are to be published on an annual report disseminated by the School of Public Health (University of Western Attica). A lag of six months to one year from the end of the AST data collection to the reporting for the respective calendar year is expected, not including the first year (2021) results that are considered preliminary and have not yet been reported. The targeted audience for these reports consists of field veterinarians, veterinary diagnostic laboratories, farming organizations and relevant governmental bodies.

#### 2.1.8. Evaluation

The pilot project was implemented for the first time in 2021. Regarding its evaluation, there were no defined performance indicators except the total number of samples screened during the year as a portion of the targeted number; this number was defined as the sample size that would allow statistically an estimation of prevalence with a 5% margin of error and a confidence interval of 95% at an infinite population (*n* = 384 for each species) [17].

### 2.2. SWOT Analysis of the Pilot Project

During the organization of the pilot project, a SWOT analysis was produced (Supplementary Material, Table S1). Nine themes are used to describe and analyze the results, as performed previously by other countries-members of the EARS-Vet Network [8]: (1) Public awareness and Policies, (2) Flexibility and Utility, (3) Data access and Sampling, (4) Data management and Analysis, (5) Harmonization, (6) Representativeness, (7) Geographical coverage, (8) Collaboration and Integrated monitoring, and (9) Sustainability and Resources.

#### 2.2.1. Strengths

The Pilot project in Greece will report annually AMR results, presenting data at trainings and lectures of the stakeholders as well as reporting survey results at an Administrative Region scale. Organizing a two-member Coordination Team of the Pilot Project inside the NAP Steering Committee allowed great flexibility and easiness in the organization of the pilot project, while at the same time, a good collaboration was achieved among various stakeholders from the private and public sector; the small country size, thus, the small number of key experts who already have experience and previous collaborations helps on this. The scope of the pilot project is narrow, and the needs of harmonization are minimal as one accredited lab is performing the analyses so far and all isolates are currently stored for future investigation. The small focus on only two bacterial pathogens and animal species theoretically makes it easy to achieve good representativeness while, in the future, the project can show great flexibility, adding more surveillance objectives according to needs and capabilities.

#### 2.2.2. Weaknesses

A weakness of the Greek pilot project is due to the lack of formalization; even though the State supports this initiative, all participation is on a voluntary basis and AMR data collection on pathogens derived from diseased animals is not enforced by any legislative framework. This creates many implications in various aspects: participation of the data providers (farmers, veterinarians, diagnostic laboratories) is not guaranteed, and their participation may be hindered by other concerns. For example, a lot of farmers are afraid of exposing inappropriate antimicrobials use during their practice and veterinarians and private diagnostic laboratories have their own conflicts of interest with their customers. In direct relation to this informality of the project, and even more important, is the limited resources availability. As the State does not financially support the project, the available financial and human resources do not allow the expansion of the project or performing in-depth additional analyses/investigations. Hence, the scope of the project is kept narrow (only two bacterial pathogens and animal species) in order to achieve a good representativeness at least for these targets.

Even though performing laboratory analysis in only one laboratory is good for some aspects (e.g., harmonization), this has a negative impact on the efforts to achieve good geographic coverage of the survey results. Moreover, AST via the disk diffusion technique is not the optimal to achieve harmonization with the results obtained from other national surveillance networks participating in EARS-Vet [8]. Data management and analysis appears also as a major weakness; data management tools are not available, raw data is basically collected on spreadsheets, making data analysis difficult and time consuming. To address this weakness temporarily, WHONET data management and the analysis program is used during the pilot project, however, it is not animal-focused and thus, a more appropriate tool needs to be developed and used in the future.

#### 2.2.3. Opportunities

Antimicrobial Resistance is considered as a high priority issue for Greek governmental policy. The Greek NAP for combating AMR (in the context of the “One Health” approach) [18] in compliance with the EU One Health Action Plan [2], supports the need to monitor AMR. These facts create optimism for sustainability, support and future expansion of projects on AMR surveillance on diseased animals. Moreover, increased public, farmers and veterinarians’ awareness on AMR encourages efforts to raise the necessary funding; the European Commission can play a significant role on this important aspect.

There is also great interest from other private diagnostic laboratories in Greece to participate in the project, however, not on a voluntary basis; financial support and governmental policies implementation would help towards this direction. The participation of more private labs will allow not only better representativeness and geographic coverage of the collected data, but also the direct expansion of the scope of the project, adding more bacterial pathogens and animal species of interest. The private labs’ network of veterinary customers can have a great positive impact on the data collection aspect. Finally, the direct connection of the Greek surveillance network to the EARS-Vet network creates huge opportunities for adopting other countries’ practices and solutions that will allow the successful implementation of the project.

#### 2.2.4. Threats

The harsh economic situation in agriculture is a direct threat to data collection; the interest to participate on a voluntary basis in the pilot project and thus, pay for sample collection and analysis is being reduced. As there is no official financial support for the project, low and decreasing staff and resource levels make the whole system too vulnerable; even minor temporary shortages in personnel and materials may completely hinder the data collection, analysis and reports. At the same time, public sector laboratories in Greece are closing and merging due to financial constraints. Even though some harmonization issues may arise if participation of more laboratories in the surveillance network is achieved, the participation of a central public sector laboratory (the national reference laboratory of AMR in Greece) will allow these problems to be addressed. Even though the increase in the animal species and pathogens targeted in the project is desired, if this is to be performed without financial support, aspects like representativeness and geographic coverage of the data collected will be significantly reduced.

## 3. Discussion

To our knowledge, this is the first presentation of an approach to organize a national monitoring system for AMR in bacterial pathogens of animals in Greece, describing system structures and operations. As in Greece, a national monitoring system is not established yet, a pilot project was launched, organized through a series of physical and online meetings chaired by MRDF; a SWOT analysis was performed to evaluate the various aspects of organization procedures and analyze all advantages and disadvantages characterizing the Greek approach to build an initial pilot project. The series of meetings among the stakeholders (farm organizations, field veterinarians, private laboratory representatives and central competent authorities) allowed the provision of a broad overview of the structure and operations of building a national monitoring system, by covering all necessary key areas.

**Figure 1:**
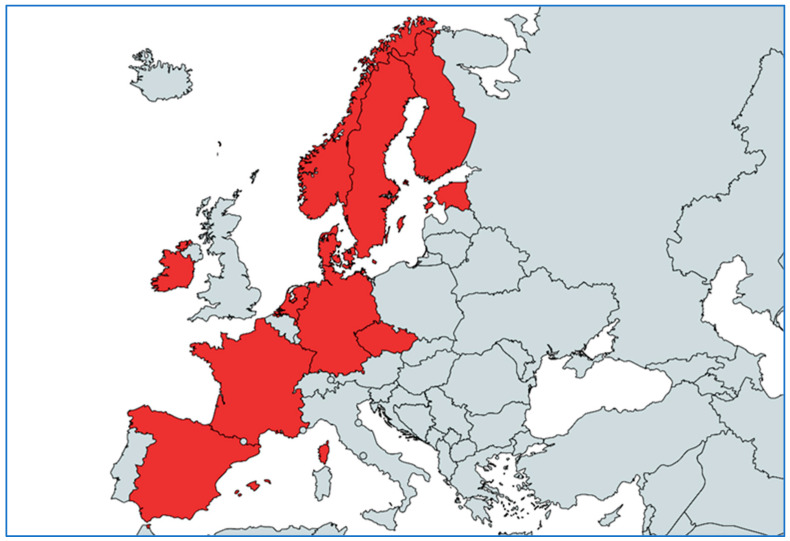
Map showing the 11 countries that reported having at least one national monitoring system for AMR in bacterial pathogens of animals. (https://www.mapchart.net/europe.html, accessed on 9 July 2023).

The pilot project organized in Greece was based on previous experience collected in other countries already implementing national monitoring systems; unfortunately, many Southern and Eastern European countries, that are characterized by similar AMR trends in the human and animal sector, do not have any national monitoring systems for AMR in bacterial pathogens derived from animals (Figure 1), demonstrating a major gap in AMR monitoring in Europe. All currently available national monitoring systems are characterized by many unique strengths and weaknesses [8]; a lesson to learn may be that each national monitoring system needs to be adapted to its national context, capacities and objectives. At the same time, some major common weaknesses are shared among many countries, e.g., the lack of appropriate data management tools and the low representativeness of the samples collected. Some important common threats are also demonstrated, such as the economic vulnerability with a regular decrease in available financial and human resources [8]. The expansion of the Greek pilot project to other targeted animal species and bacterial pathogens of interest, the addition of more private laboratories to the data providers involved and the efforts to create a legal framework that will promote the participation of stakeholders in the system are key aspects that should be prioritized in the future.

Even though the Greek pilot project (the forerunner to our national monitoring system) did not undergo any evaluation using a validated tool [19], a major positive characteristic was demonstrated: a collaboration between the public and the private sector was achieved. This public–private partnership constitutes a significant opportunity to develop a broad monitoring system where private stakeholders (farm industry, veterinary practitioners and private diagnostic laboratories) will share related AMR data with the public competent authorities, creating the potential to fully understand all aspects of AMR emergence and trends, the epidemiological links among animal, human and environmental sectors and propose appropriate interventions to mitigate public health impacts and raise public awareness. It will be unfortunate if this opportunity is lost due to the major threat being reported by all national monitoring systems, the economic vulnerability. As the first pilot-testing results of the EARS-Vet surveillance network are disseminated [20], the resources (financial, human, material) support of the network will ensure the sustainability needed to protect Public Health in a One Health holistic approach.

## 4. The Approach Implemented

As a first step, a survey occurred from 2018 to 2020 [8]; EU-JAMRAI stakeholders from 27 EU and European Economic Area (EEA) countries—Greece included, were contacted and asked if a national monitoring system for AMR in bacterial pathogens derived from animals was in place in their country. A national monitoring system for AMR in bacterial pathogens of animals was defined as any system that is collecting and regularly analyzing AST results produced on bacterial isolates from clinical samples of animals that can be considered as having a national coverage with no criteria being established on geographic data representativeness or scope of bacterial and animal species.

In the European Union, 11 countries reported having a national monitoring system for AMR in bacterial pathogens of animals: the Czech Republic, Denmark, Estonia, Finland, France, Ireland, Germany, the Netherlands, Norway, Spain and Sweden. Stakeholders from Denmark, the Netherlands and Sweden reported having more than one monitoring system in their countries (Figure 1).

As Greece was one of the countries that did not have a national monitoring system in place, an initiative was established by competent authorities as a next step to determine the feasibility of organizing a relevant network.

At the start, a first kick-off meeting with the scope “Identification of the feasibility in building a monitoring system of AMR surveillance in diseased animals in Greece” was announced by the School of Public Health of Greece (EU-JAMRAI Competent Authority in Greece); several Greek Representatives were invited to participate. The representatives were selected based upon their previous experience and cooperation with the School of Public Health of Greece on the subject of AMR surveillance on the veterinary sector. Participation of representatives from the human and the environmental sector with experience on the AMR public health issue was pursued as well, e.g., the co-authors of the National Action Plan (NAP), published in 2019, for the period 2019–2023, to tackle AMR [18]. No representation was pursued, at this stage, of the pharmaceutical agencies (commercial sector).

The Greek Representatives were decided to be as follows:

From the Academic Sector: (a) Department of Public Health Policy, School of Public Health (University of West Attica), (b) Hygiene of Food of Animal Origin and Veterinary Public Health School of Veterinary Medicine (Aristotle University of Thessaloniki), (c) Laboratory of Hygiene of Food of Animal Origin (Faculty of Veterinary Science, University of Thessaly), (d) Faculty of Medicine (Aristotle University of Thessaloniki), (e) Department of Animal Production (School of Animal Bioscience, Agricultural University of Athens).

From the technical sector: (a) Department of Veterinary Drugs, Residues and Veterinary Supplies & Veterinary Centre of Athens—National Reference Laboratory for AMR (Ministry of Rural Development & Food), (b) Hellenic Pasteur Institute [National focal point of the World Health Organization (WHO) for AMR and EARS-Net/Global Antimicrobial Resistance Surveillance and Use System (GLASS)], (c) Hellenic Center for Disease Control and Prevention (currently National Organization of Public Health—EODY)—Central Public Health Laboratory (Ministry of Health), (d) Special Water Secretariat (Ministry of Environment and Energy), (e) Asclepius One Health platform (Non-Profit Organization)—Chatham House (International affairs think tank).

From the political sector: (a) Ministry of Rural Development & Food (consultant of the Minister’s Cabinet).

Upon a request to explore the possibility of building a European Antimicrobial Resistance Surveillance network in Veterinary medicine (EARS-Vet) according to the EU JAMRAI Task 7.4.2 [21] in Greece, a call (via email exchanges) was initiated by the School of Public Health of Greece—EU JAMRAI Competent Authority in Greece to all previously referred stakeholders. During the kick-off meeting (a one-day physical meeting that took place in 2019), an EARS-Vet coordinator presented the current situation of the existing national surveillance of AMR systems in diseased animals in Europe, shared current experiences and gave specific examples of implemented systems. After this presentation, open discussion followed on what the participants surmise about the idea of building a national surveillance network of AMR in diseased animals. Scenarios were further discussed on how they would proceed if a monitoring system was to be built in Greece. Scientific advice was finally provided by the coordinator of the EARS Vet.

After this first kick-off meeting, the Ministry of Rural Development & Food (MRDF) defined a Coordination Team to organize the next steps. In Greece, there is a regulated system—a National Action Plan (NAP), published in 2019, for the period 2019–2023, to tackle AMR [18]. In accordance with the NAP, a Steering committee has been established, based in the MRDF—State Veterinary Administration (General Directorate of Veterinary Services) named as the “Steering Committee for the monitoring and implementation of the ‘National Action Plan to combat Antimicrobial Resistance (AMR) in the context of One—Health’ at the veterinarian sector”. The Coordination Team consisted of two members of the Steering Committee, one of which was defined as the coordinator for the pilot project to be implemented (see below). The main duties of the Coordination Team were to specify what procedures should be followed to build a monitoring system of AMR surveillance in diseased animals in Greece. In practice, the Coordination Team should combine its experience/knowledge in AMR against bacterial pathogens of animals, in compliance with a common methodology across the EU JAMRAI participating countries, with relevant feedback from all stakeholders to deliver a broad overview of the structure and operations of a national monitoring system, covering all important areas; not limited to microbiology, but also funding, regulation, institutional organization, procedures, laboratory networks, communication and evaluation.

The Coordination Team took a first decision as a next step, based on its experience/knowledge, that the first pilot project should be focused on cattle and swine samples. After this, the team sought help/feedback from a series of stakeholders related to the cattle and swine industry and research stakeholders: farm organizations, farm veterinarians, private laboratories representatives and central competent authorities. To achieve this, a call (via email) for a series of physical presence/teleconference meetings was made to all of the above-mentioned Greek stakeholders. During those meetings, representatives were asked to answer a semi-structured questionnaire covering key areas as suggested by the EARS-Vet [7] in order to define the combinations of animal species, bacterial species and antimicrobials which would be the most relevant and feasible to monitor at a national level in compliance with the “One Health” approach.

Finally, after collecting all the relevant feedback from the various stakeholders, a one-day physical presence meeting took place in 2020 where relevant stakeholders and the members of the Coordination Team participated. During that meeting, all participants were asked to answer a semi-structured questionnaire based on the one designed by EARS-Vet [8] covering the following key areas: (i) political and financial support, (ii) monitoring objectives, (iii) central institutional organization, (iv) laboratory network, (v) monitoring procedures—monitoring data, (vi) laboratory techniques, (vii) communication, and (viii) evaluation.

The Ministry of Rural Development and Food eventually launched a pilot project entitled “Trying to build our national surveillance system for the monitoring of the Antimicrobial Resistance in diseased animals (cattle and swine)”.

## 5. Conclusions

The Greek pilot project on AMR surveillance in diseased animals showcases several strengths, including regular reporting, a flexible Coordination Team and successful collaboration among stakeholders. The focused scope and minimal harmonization contributes to its representativeness and its adaptable nature allows for future expansion. However, challenges such as the lack of formalization, limited resources and difficulties in data management hinder its full potential. Nevertheless, opportunities arise from the government’s prioritization of AMR, increased public awareness and potential support from the European Commission. Nonetheless, economic challenges in agriculture and the need for financial backing pose threats to its sustainability and data quality. Emphasizing the One Health approach is essential in addressing these weaknesses and leverage opportunities to enhance AMR surveillance in Greece, thereby safeguarding public health, animal health and the environment collaboratively.

## Figures and Tables

**Table 1:** Animal species, production types, specimens and bacterial species to be covered by the pilot project—Antimicrobial Resistance Surveillance network in Veterinary medicine in Greece.

Animal Species	Production Type	Specimens	Bacterial Species
Cattle	Dairy Cows (>18 months of age)	Milk	*Staphylococcus aureus*
Swine	Post-weaning piglets (fattening pigs)	Faeces	*Escherichia coli*

**Table 2:** Bacterium-antimicrobial combinations included in the pilot project—Antimicrobial Resistance Surveillance network in Veterinary medicine in Greece.

Bacterial Species	Antimicrobial Group	Antimicrobial Agents
*Staphylococcus aureus*	Penicillin	Penicillin
	Methicillin resistance	Cefoxitin
	Fluoroquinolones	Enrofloxacin
	Lincomycin	Lincomycin
	Gentamicin	Gentamicin
*Escherichia coli*	Aminopenicillins	Ampicillin
	Third generation cephalosporins	Ceftiofur
	Fluoroquinolones	Enrofloxacin
	Tetracyclines	Tetracycline
	Colistin	Colistin
	Gentamicin	Gentamicin

**Table 3:** Specific relevant indications of the antibiotics targeted in the Greek pilot project.

Animal Species	Antimicrobial Agents	Relevant Indications
Cattle	Penicillin	Broad-spectrum antibiotic that works effectively against many gram-positive bacteria. It is commonly used to treat infections caused by bacteria like *Staphylococcus* spp. in animals.
	Cefoxitin	Broader spectrum of activity compared to penicillin. It is often used to treat infections caused by both gram-positive and gram-negative bacteria. Cefoxitin is particularly valuable in cases of mixed infections or when bacteria show resistance to penicillin.
	Enrofloxacin	Broad spectrum of activity against both gram-negative and some gram-positive bacteria. It is commonly used in veterinary medicine to treat mastitis in cows and other animals.
	Lincomycin	Effective against certain gram-positive bacteria, including some strains of *Staphylococcus*. It is one of the antibiotics commonly used to treat mastitis in cows and other animals.
	Gentamicin	Primarily used to treat severe infections caused by gram-negative bacteria. It can also be used in combination with other antibiotics to treat resistant infections.
Swine	Ampicillin	Broad-spectrum penicillin-type antibiotic. It is commonly used to treat gastrointestinal tract infections in animals.
	Ceftiofur	Broad spectrum of activity against both gram-positive and gram-negative bacteria. In cases of diarrhea in pigs, where bacterial infections are the underlying cause, ceftiofur may be prescribed by a veterinarian.
	Enrofloxacin	Broad spectrum of activity against both gram-negative and some gram-positive bacteria. It is commonly used in veterinary medicine, including for the treatment of diarrhea in pigs.
	Tetracycline	Broad-spectrum antibiotic that works against a wide range of bacteria, both gram-positive and gram-negative. It is particularly useful in treating gastrointestinal infections in pigs, as it can target bacteria commonly associated with diarrhea, such as *Escherichia coli* and *Salmonella* spp.
	Colistin	Antibiotic of last resort used to treat severe infections caused by multidrug-resistant gram-negative bacteria. It is used sparingly and, as a last resort, due to concerns about the development of resistance.
	Gentamicin	Primarily used to treat severe infections caused by gram-negative bacteria. It can also be used in combination with other antibiotics to treat resistant infections.

## Data Availability

Data are contained within this article or Supplementary Material.

## Supplementary Materials

The following supporting information can be downloaded at https://www.mdpi.com/article/10.3390/antibiotics12091442/s1, Table S1: SWOT Analysis of the Pilot Project Organization in Greece.
